# Wisdom before knowledge--appraisal of the scientific evidence used to develop guidelines for the diagnosis of arrhythmogenic right ventricular dysplasia

**DOI:** 10.1186/1532-429X-15-S1-E123

**Published:** 2013-01-30

**Authors:** Tessa S Cook, Saurabh Jha

**Affiliations:** 1 Radiology, Hospital of the University of PA, Philadelphia, PA, USA

## Background

Guidelines are an important vehicle in the era of evidence-based medicine and diagnosis. They represent a culmination of expert opinions and the best available evidence. There can be a tendency for practitioners to view and apply guidelines as the absolute truth. However, it is important to remember that guidelines are only as robust as the strength of the studies that are used in their development.

Arrhythmogenic right ventricular dysplasia (ARVD) is a relatively rare disease that primarily affects the right ventricle and can be potentially fatal if left untreated. Cardiac MRI (CMR) plays a supportive role in the diagnosis. A task force was established in 1994 and another convened in 2009, in order to develop guidelines for the diagnosis of ARVD. The aim of this exhibit is to critically appraise the studies that were used to classify the findings on CMR into major and minor criteria for the diagnosis of ARVD. The studies will be critiqued particularly for statistical biases and the potential for overdiagnosis.

## Methods

The citations pertaining to CMR in the published guidelines from 1994 and 2009 have been appraised using the QUADAS method. In addition, a decision tree (Tree Age Pro 2012) was used to study the potential for overdiagnosis using CMR at various pre-test probabilities, test sensitivities and specificities.

## Results

The studies used to develop the revised task force criteria for ARVD present several biases for discussion including incorporation bias, verification bias, double gold standard bias and spectrum bias, as well as the problems associated with an unreliable gold standard. The revised guidelines have resulted in a trade off between sensitivity and specificity of uncertain magnitude. Furthermore, there is considerable potential for overdiagnosis of ARVD.

## Conclusions

Even though the new task force guidelines provide specific quantitative cutoffs for the diagnosis of ARVD using measures extracted from CMR exams, there remain significant statistical biases in the studies used to derive the guidelines. Imagers are advised to use the criteria set by the task force in conjunction with the pre-test probability of ARVD when rendering an interpretation on CMR done for suspected ARVD.

## Funding

None

